# Factors associated with the need of parenteral nutrition in critically ill patients after the initiation of enteral nutrition therapy

**DOI:** 10.3389/fnut.2023.1250305

**Published:** 2023-08-24

**Authors:** Juan Carlos Lopez-Delgado, Lluís Servia-Goixart, Teodoro Grau-Carmona, Luisa Bordeje-Laguna, Esther Portugal-Rodriguez, Carolina Lorencio-Cardenas, Paula Vera-Artazcoz, Laura Macaya-Redin, Juan Francisco Martinez-Carmona, Judith Marin Corral, Jose Luis Flordelís-Lasierra, Carlos Seron-Arbeloa, Maravillas de las Nieves Alcazar-Espin, Elisabeth Navas-Moya, Sara Aldunate-Calvo, Beatriz Nieto Martino, Itziar Martinez de Lagran

**Affiliations:** ^1^Hospital Clinic of Barcelona, Barcelona, Spain; ^2^Departament d’Infermeria Fonamental i Médico-Quirúrgica, School of Nursing, University of Barcelona, Barcelona, Spain; ^3^University Hospital Arnau de Vilanova, Lleida, Spain; ^4^Lleida Institute for Biomedical Research (IRBLleida), Lleida, Spain; ^5^University Hospital October 12, Madrid, Spain; ^6^Research Institute Hospital 12 de Octubre, Madrid, Spain; ^7^Hospital Germans Trias i Pujol, Badalona, Spain; ^8^Hospital Clínico Universitario de Valladolid, Valladolid, Spain; ^9^Doctor Josep Trueta Girona University Hospital, Girona, Spain; ^10^Hospital Santa Creu i Sant Pau, Barcelona, Spain; ^11^Complejo Hospitalario de Navarra, Pamplona, Spain; ^12^Regional University Hospital of Malaga, Málaga, Spain; ^13^Hospital del Mar, Parc de Salut Mar, Barcelona, Spain; ^14^Hospital General San Jorge, Huesca, Spain; ^15^Hospital General Universitario Morales Meseguer, Murcia, Spain; ^16^Vall d'Hebron University Hospital, Barcelona, Spain; ^17^Fuenlabrada University Hospital, Madrid, Spain; ^18^Mataró Hospital, Barcelona, Spain

**Keywords:** nutrition therapy, intensive care unit, enteral nutrition, parenteral nutrition, gastrointestinal dysfunction

## Abstract

**Background and aims:**

Despite enteral nutrition (EN) is the preferred route of nutrition in patients with critical illness, EN is not always able to provide optimal nutrient provision and parenteral nutrition (PN) is needed. This is strongly associated with gastrointestinal (GI) complications, a feature of gastrointestinal dysfunction and disease severity. The aim of the present study was to investigate factors associated with the need of PN after start of EN, together with the use and complications associated with EN.

**Methods:**

Adult patients admitted to 38 Spanish intensive care units (ICUs) between April and July 2018, who needed EN therapy were included in a prospective observational study. The characteristics of EN-treated patients and those who required PN after start EN were analyzed (i.e., clinical, laboratory and scores).

**Results:**

Of a total of 443 patients, 43 (9.7%) received PN. One-third (29.3%) of patients presented GI complications, which were more frequent among those needing PN (26% vs. 60%, *p* = 0.001). No differences regarding mean energy and protein delivery were found between patients treated only with EN (*n* = 400) and those needing supplementary or total PN (*n* = 43). Abnormalities in lipid profile, blood proteins, and inflammatory markers, such as C-Reactive Protein, were shown in those patients needing PN. Sequential Organ Failure Assessment (SOFA) on ICU admission (Hazard ratio [HR]:1.161, 95% confidence interval [CI]:1.053–1.281, *p* = 0.003) and modified Nutrition Risk in Critically Ill (mNUTRIC) score (HR:1.311, 95% CI:1.098–1.565, *p* = 0.003) were higher among those who needed PN. In the multivariate analysis, higher SOFA score (HR:1.221, 95% CI:1.057–1.410, *p* = 0.007) and higher triglyceride levels on ICU admission (HR:1.004, 95% CI:1.001–1.007, *p* = 0.003) were associated with an increased risk for the need of PN, whereas higher albumin levels on ICU admission (HR:0.424, 95% CI:0.210–0.687, *p* = 0.016) was associated with lower need of PN.

**Conclusion:**

A higher SOFA and nutrition-related laboratory parameters on ICU admission may be associated with the need of PN after starting EN therapy. This may be related with a higher occurrence of GI complications, a feature of GI dysfunction.

**Clinical trial registration:**

ClinicalTrials.gov: NCT03634943.

## 1. Introduction

Appropriate nutrition therapy of severely ill patients admitted to intensive care units (ICUs) aims at avoiding malnutrition of primarily well-nourished patients and preventing further deterioration of preexisting malnutrition. Nutrition therapy with optimal delivery of nutrients provides the substrates to preserve organ function, helps to modulate the inflammatory response to surgery, trauma, or any severe disease, and optimizes the metabolic status, all of which may ultimately contribute to improvement in clinical patient outcomes (1, 2). Malnutrition has been shown to be a significant prognostic factor for mortality, length of stay, infection rates, and duration of mechanical ventilation (3, 4).

However, performing an adequate nutrition therapy remains difficult because critically ill suffer from pathophysiological alterations in metabolism. During the acute phase of critical illness (i.e., first 48–72 h of ICU admission), there is an increased demand of metabolic substrates, especially glucose, caused by higher resting energy expenditure needs, which are mainly obtained from the stimulation of gluconeogenesis (5–8). Furthermore, exogenous substrates no longer inhibit the production of glucose by gluconeogenic organs (e.g., liver), and nutrition therapy should be progressive during the acute phase to avoid the detrimental effects of overfeeding (i.e., >110% of energy demands) over morbidity (e.g., longer length of mechanical ventilation) and even mortality (7, 8). At the same time, glycogen reserves in the liver do not last long (i.e., <24 h) and patients suffer from muscle catabolism since amino acids are main source to preserve gluconeogenesis, leading to protein depletion and sarcopenia, which is linked with worst outcomes (9).

Although nutritional therapy is an integral part in the care of ICU patients and clinical practice recommendations for nutrition in critically ill patients have been published by different guidelines (10–13), macronutrient nutrition targets (i.e., caloric and protein) are frequently not achieved in routine daily conditions (14, 15). For example, a recent large observational study showed that almost 20 and 35% of ICU patients do not receive adequate delivery of protein targeted needs (16). These findings are also quite similar to previous studies (17, 18). Indeed, the benefits of nutrition therapy in ICUs are related to several factors, including the delivery route, initiation time, macronutrient dose, patient’s nutritional risk, duration of ICU stay, or severity of critical illness.

Enteral nutrition (EN) is the preferred nutritional route for the majority of critically ill patients, with advantages over parenteral nutrition (PN), such as cost-effectiveness, efficient use of nutrients, maintenance of gastrointestinal barrier or support of intestinal immunological function, but there is a large variation in how nutritional therapy is provided clinically, which ultimately difficult macronutrient targets (10, 11). Moreover, barrier factors influencing enteral feeding include delay in the start of EN, low infusion rate, lack of standardized and failure to follow EN protocols, disruptions to EN (e.g., diagnostic testing, accidental pull-out of nasogastric tubing, gastrointestinal intolerance), insufficient dietitian coverage, and prioritize other aspects of patient care over nutrition (19–22). Despite these factors can be overcome by improving adherence to guidelines, there are some ICU patients not able to tolerate prescribed EN due to the occurrence of gastrointestinal (GI) complications (e.g., high gastric residual volumes, diarrhea, vomiting), leading to the lack of optimal nutrient provision (23, 24). In consequence, this suboptimal EN intake may worsen nutritional status, which is associated with higher complications (e.g., infections) and higher mortality (25). At the same time, it is important to remark that the occurrence of GI complications and EN intolerance are both features of GI dysfunction (25).

The use of PN after the initiation of EN could help to improve nutritional intake and mitigate the development of malnutrition in ICU patients who do not tolerate prescribed EN (26). Moreover, the use of PN has also been associated with disease severity and higher mortality, suggesting that it is a surrogate marker of GI dysfunction (27, 28). In a previous nationwide evaluation of nutritional practices in ICU patients admitted to 38 Spanish ICUs and focused on ICU mortality, patients who needed PN after starting EN were found to be a high-risk group for mortality (23).

The main aim of the present study was to explore the potential of different factors (i.e., clinical variables, laboratory markers, and nutritional and ICU scores) to be associated with the need of PN after starting EN, especially those recorded on ICU admission or during the early stage. We hypothesized that this may be helpful to identify early those patients that may need PN after starting EN and to prescribe earlier PN, avoiding suboptimal nutrition intake, and the development of malnutrition during ICU stay, which ultimately may prevent negative effects of GI dysfunction. The present research may be also helpful to reflect the difficulty of achieving macronutrient targets only with enteral route (i.e., EN) under certain clinical conditions in critically ill. We also aimed to describe the use and GI complications, especially those associated with EN, together with differences in nutrition therapy between patients receiving EN only and those who need PN after starting EN were examined.

## 2. Materials and methods

### 2.1. Study design and participants

Between April and July 2018, a nationwide prospective observational study was conducted in which 38 ICUs throughout Spain participated (We shown in Supplementary Table S1 location of participating hospitals). All adult patients aged 18 years or older were consecutively included in the study providing that they need artificial nutrition support (EN or PN or both) for more than 48 h and a length of stay in the ICU of at least 72 h. Patients able to feed orally were excluded. Patients admitted to the ICU for postoperative recovery or ICU monitoring without the needing specific treatment for organ support (e.g., non-invasive mechanical ventilation or vasopressors) were also excluded. Only patients who required initial EN on ICU admission were included in the present analysis. Results of the present research corresponds to a planned subanalysis about the insights of the use of EN in the ENPIC study (ClinicalTrials.gov: NCT03634943). Despite the observational nature of the study, participating ICUs were required to have at least clinical nutrition practices that included the use of a local nutritional protocol in compliance with current guidelines or in the absence of this, the involvement of a health care professional specialized in artificial nutrition therapy (15). Participating sites and investigators were encouraged to follow recommendations for the feeding route delivery and management for giving optimal nutrition therapy in ICU patients (see Supplementary Figure S1) (13, 15). It is important to highlight that all participants were interested in clinical nutrition and are members of the Metabolism & Nutrition Working Group the Spanish Society of Intensive Care Medicine (SEMICYUC) and/ or Critical Care Working Group from the Spanish Society of Metabolism & Clinical Nutrition (SENPE).

### 2.2. Ethics statement

The study was approved by the Clinical Research Ethics Committee of Hospital Universitari de Bellvitge (Barcelona, Spain) as a central institutional review board (code PR401/17). The need for informed consent was waived due to the observational design of the study and collection of data from an anonymous centralized database.

### 2.3. Data collection and definitions

For each patient, the following data were collected: demographics; body mass index; type of patient (medical, trauma, surgery); comorbidities; Acute Physiology and Chronic Health Evaluation (APACHE) II score; Simplified Acute Physiology Score (SAPS) II score; Sequential Organ Failure Assessment (SOFA) score on ICU admission; modified Nutrition Risk in the Critically II (mNUTRIC) score; details of nutritional therapy including time of initiation of EN, mean energy and protein intakes until ICU discharge or for a maximum of 14 days; laboratory data; GI (or EN-related) complications (i.e., high gastric residual volume, vomiting, aspiration, diarrhea, mesenteric ischemia); and outcomes during ICU stay, which included mechanical ventilation and days on mechanical ventilation, vasoactive drug support, renal replacement therapy (RRT), respiratory tract infection, catheter-related bloodstream infection, length of stay in the ICU and in the hospital, and 28-day mortality. We registered mNUTRIC score as dichotomous variable to evaluate nutritional risk: a mNUTRIC score ≥ 5 on ICU admission was considered a higher nutritional risk when compared with a score 0–5. The type of nutritional therapy included two subgroups: EN only and EN-PN (i.e., patients who received EN initially followed by PN).

### 2.4. Outcomes

The study outcomes included the difference between EN and EN-PN groups in GI complications, mechanical ventilation, duration of mechanical ventilation, use of vasoactive drugs, RRT, respiratory and catheter-related infections, length of ICU and hospital stay, and 28-mortality.

### 2.5. Statistical analysis

Categorical data are expressed as frequencies and percentages, and continuous data as mean and standard deviation (SD) or median an interquartile range (IQR) (25th-75th percentile). Categorical data were compared with the chi-square test or the Fisher’s exact test, and quantitative data with the Student’s *t* test or the Mann–Whitney *U* test according to the conditions of application. Survival analysis was performed using the Kaplan–Meier method with the Log-Rank test to assess the need of PN over time.

Variables independently associated with the need of supplemental PN were assessed using an adjusted multiple stepwise Cox regression analysis. Variables were included in the model if they had a *p* value of <0.2 in the univariate analysis. Subsequent multivariate analysis was conducted using an adjusted multiple stepwise Cox regression analysis adjusted by age, patient type (e.g., medical, surgical, or trauma), illness severity (e.g., APACHE score), length of nutritional therapy, and data for which there were significant differences in baseline characteristics between both subgroups. The size difference between EN and EN-PN groups (i.e., number of patients) and time of initiation of PN was also considered for adjusting. Statistical significance was set at *p* < 0.05. The IBM SPSS version 20.0 (IMB Corp., Armonk, NY, United States) was used for the analysis of data.

## 3. Results

**Figure 1:**
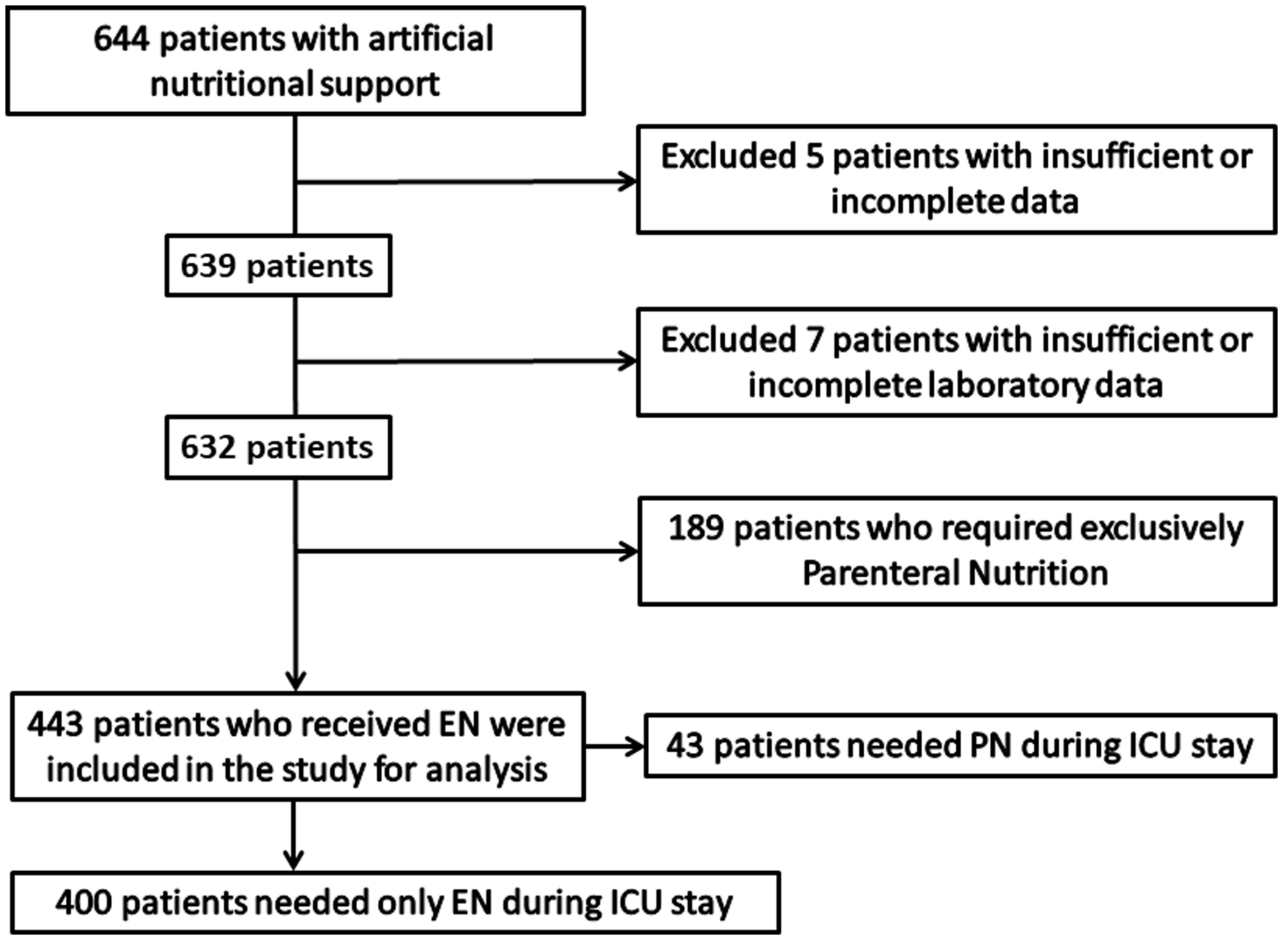
Flow chart of the study population.

During the study period, 644 ICU patients received artificial nutrition support, but 201 patients were excluded because of incomplete data collection (*n* = 13) or use of initial PN (*n* = 189). Of the remaining 443 patients treated with EN, 400 (90.3%) received EN exclusively and 43 (9.7%) were treated with EN and PN. The study flow chart is shown in Figure 1.

Most of the patients (*n* = 426, 96.2%) received EN by means of nasogastric tube, whereas a few patients received EN by the transpyloric route (*n* = 6, 1.3%) or via a surgical ostomy (*n* = 11, 2.5%). Almost half of the patients (*n* = 216, 48.8%) made a switch from EN to oral nutrition before ICU discharge. No patient included in this analysis received any oral supplements.

**Figure 2:**
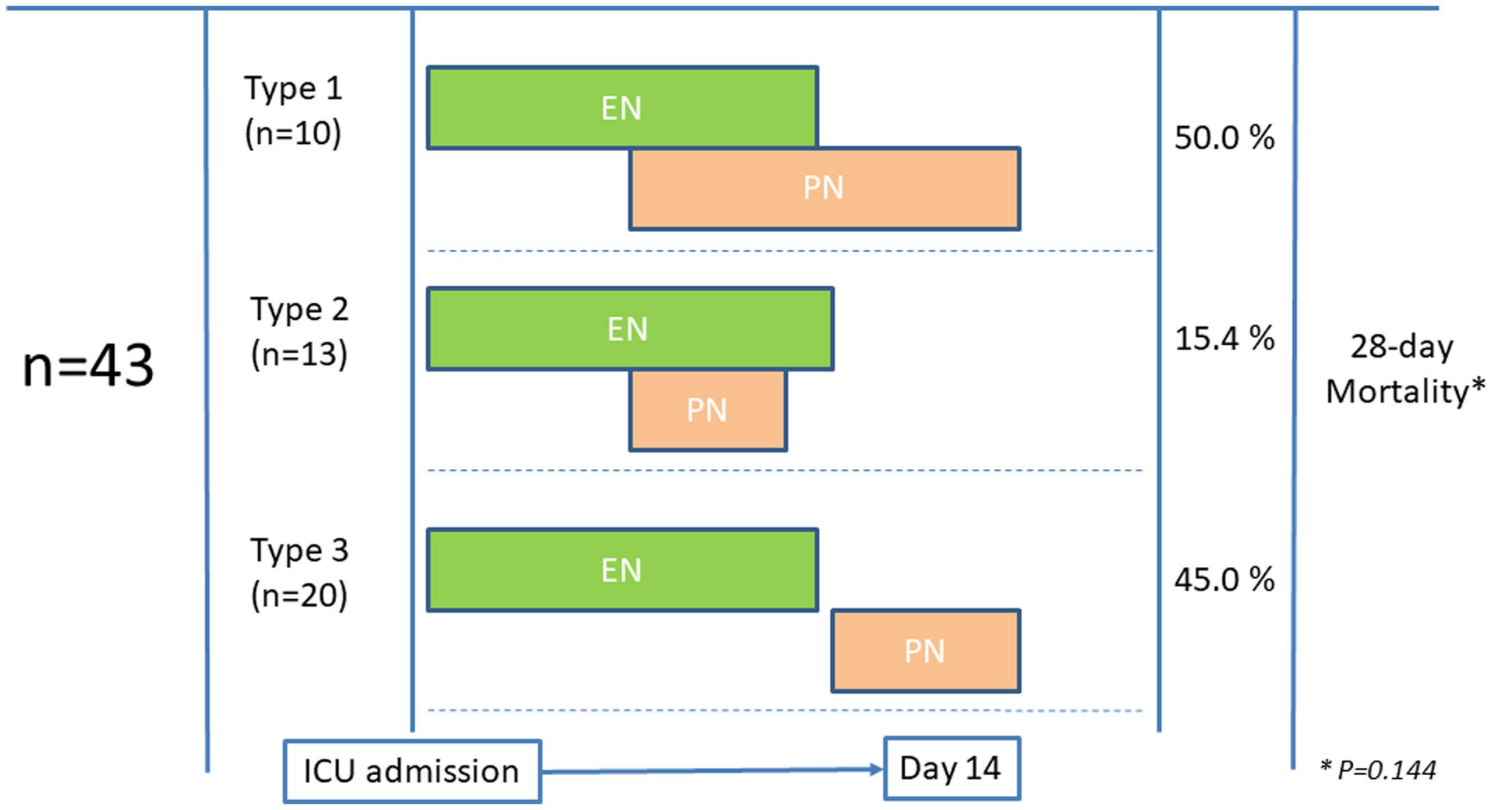
Patterns of administration of parenteral nutrition (PN) in patients who initially received enteral nutrition (EN).

In the group of 43 patients with EN-PN, the indication of PN was related to difficulties in achieving sufficient nutritional requirements (*n* = 38, 88.4%) (i.e., <60% of macronutrient target (2, 13)), mainly due to paralytic ileus (*n* = 23, 53.5%). Very few patients received PN due to severe acute pancreatitis (*n* = 3, 6.9%) and small bowel fistula (*n* = 2, 4.6%). PN was usually administered through a central venous line, except in 4 (9.3%) patients in which a peripherally inserted central catheter was used. All patients from EN-PN received prokinetics (i.e., metoclopramide, erythromycin or both) before the indication of PN. As shown in Figure 2, a total of 23 (53.5%) patients received supplemental PN in addition to EN and 20 (46.5%) received PN following discontinuation of EN. Nutritional therapy was given for a median duration of 15 days (range from 6 to 15 days), and PN was given between day 3 and 10 after ICU admission in all cases.

The clinical characteristics, nutritional support, and outcomes of the patients receiving EN and EN-PN are shown in Table 1. Most patients were admitted due to medical illnesses (71.3%), received early EN (i.e., <48 h) support (75.4%) and a higher proportion of patients were at risk of malnutrition based on mNUTRIC score (43.34%). SOFA (on ICU admission) was higher in those who needed PN (Hazard ratio [HR]:1.161, 95% confidence interval [CI]:1.053–1.281, *p* = 0.003) and those patients with low SOFA (from 0 to 6) showed lower need of PN (HR:0.283, 95% CI:0.120–0.668, *p* = 0.004). The mNUTRIC score was higher in those who need PN (HR:1.311, 95% CI:1.098–1.565, *p* = 0.003).

**Table 1:** Clinical characteristics, nutritional support, and outcomes of ICU patients receiving enteral nutrition (EN) and enteral nutrition with parenteral nutrition (EN-PN) during ICU stay.

	All patients (*n* = 443)	EN (*n* = 400)	EN-PN (*n* = 43)	*P* value
Baseline characteristics and comorbidities				
Age, years, mean ± SD	60.67 ± 15.26	60.72 ± 15.45	60.23 ± 13.56	*0.84*
Sex, males	298 (67.3%)	267 (66.7%)	31 (72.1%)	*0.60*
BMI, kg/m^2^, mean ± SD	28.07 ± 6.20	28.17 ± 6.32	27.12 ± 4.92	*0.29*
Hypertension	187 (42.2%)	170 (42.5%)	17 (39.5%)	*0.74*
Diabetes mellitus	116 (26.2%)	103 (25.7%)	13 (30.2%)	*0.58*
Chronic obstructive pulmonary disease	81 (18.3%)	72 (18.0%)	9 (20.9%)	*0.67*
Acute myocardial infarction	66 (14.9%)	59 (14.7%)	7 (16.3%)	*0.82*
Chronic liver disease	22 (5.0%)	22 (5.5%)	0	*0.15*
Chronic renal failure	45 (10.2%)	42 (10.5%)	3 (7.0%)	*0.60*
Immunosuppression	48 (10.8%)	42 (10.5%)	6 (13.9%)	*0.44*
Neoplasia	69 (15.6%)	61 (15.2%)	8 (18.6%)	*0.51*
Type of patient				
Medical	316 (71.3%)	285 (71.2%)	31 (72.1%)	*0.99*
Trauma	63 (14.2%)	59 (14.7%)	4 (9.3%)	*0.39*
Surgery	64 (14.4%)	56 (14.0%)	8 (18.6%)	*0.28*
APACHE II	20.28 ± 7.89	20.04 ± 7.85	22.49 ± 8.03	*0.05*
SAPS II	48.62 ± 17.27	48.37 ± 17.41	51.03 ± 15.94	*0.36*
SOFA (ICU admission)	7.24 ± 3.24	7.09 ± 3.17	8.65 ± 3.54	***0.003***
Patient with malnutrition (based on SGA)	154 (34.8%)	138 (34.5%)	16 (37.2%)	*0.73*
mNUTRIC Score	4.04 ± 2.18	3.96 ± 2.17	4.81 ± 2.17	***0.016***
Patient at risk (based on mNUTRIC)	192 (43.3%)	165 (41.2%)	27 (62.8%)	***0.021***
Nutritional support				
Time of initiation of EN (h)	37.15 ± 30.85	36.40 ± 31.31	44.15 ± 25.49	*0.12*
Early nutritional support (<48 h)	334 (75.4%)	308 (77.0%)	26 (60.5%)	***0.024***
Mean Kcal/kg/day*	14.59 ± 5.58	14.50 ± 5.60	15.46 ± 5.31	*0.28*
Mean g protein/kg/day*	0.77 ± 0.34	0.76 ± 0.34	0.83 ± 0.28	*0.24*
Gastrointestinal complications				
Any complication	130 (29.3%)	104 (26.0%)	26 (60.5%)	***0.001***
Gastric residual volume (<500 mL)	62 (14.0%)	46 (11.5%)	16 (37.2%)	***0.01***
Diarrhea	43 (9.7%)	35 (8.7%)	8 (18.6%)	*0.10*
Vomiting	5 (1.1%)	5 (1.2%)	0	*0.60*
Mesenteric ischemia	7 (1.6%)	3 (0.7%)	4 (9.3%)	***<0.001***
Outcomes				
Mechanical ventilation	431 (97.3%)	391 (97.7%)	40 (93.0%)	*0.10*
Days on mechanical ventilation	14.00 ± 14.59	13.23 ± 13.94	21.50 ± 18.35	***0.001***
Vasoactive drug support	328 (74.0%)	296 (74.0%)	32 (74.4%)	*0.99*
Renal replacement therapy	58 (13.1%)	41 (10.2%)	17 (39.5%)	***<0.001***
Respiratory tract infection	110 (24.8%)	102 (25.5%)	8 (18.6%)	*0.36*
Catheter-related infections	28 (6.3%)	26 (6.5%)	2 (4.6%)	*1*
ICU stay, days, mean ± SD	19.09 ± 16.63	18.58 ± 16.23	23.88 ± 19.50	***0.047***
Hospital stay, days, mean ± SD	35.04 ± 29.65	34.60 ± 29.80	39.20 ± 28.15	*0.34*
28-day mortality	115 (26.0%)	99 (24.7%)	16 (37.2%)	*0.09*

SD, standard deviation; APACHE II, Acute Physiology and Chronic Health Disease Classification System II; SAPS II, Simplified Acute Physiology Score II; SOFA, Sequential Organ Failure Assessment; SGA, Subjective Global Assessment; mNUTRIC, modified Nutrition Risk in the Critically Ill. *During the whole administration of nutritional support or at least the first 14 days of nutritional support. *P* significant value is written in bold.

**Figure 3:**
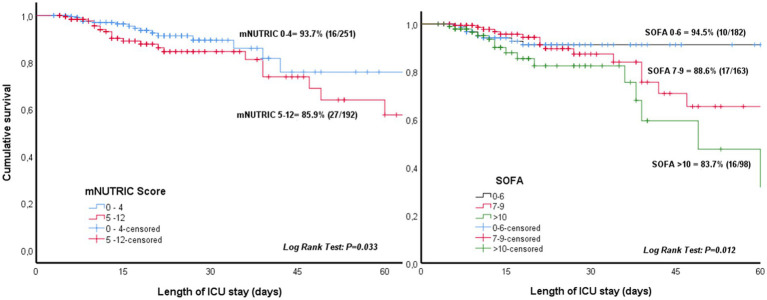
Need of parenteral nutrition during ICU admission according to the sequential organ failure assessment (SOFA) score on ICU admission and modified nutrition risk in the critically Ill (mNUTRIC) score.

The percentage of patients who needed PN after initiation of EN varied significantly according to SOFA score on ICU admission (5.5% for SOFA score 0–6, 11.4% for SOFA score 7–9, and 16.3% for SOFA score ≥ 10, *p* = 0.013). Also, the percentage of patients requiring PN were higher for higher nutritional risk, which is a mNUTRIC score ≥ 5 (14.1%) as compared with a score 0–5 (6.3%) (*p* = 0.021). Kaplan–Meier survival analysis also showed higher need of PN with higher SOFA and mNUTRIC scores during ICU admission (Figure 3).

Patients in the EN group received early nutritional therapy (i.e., <48 h) more frequently than those in the EN-PN group (77% vs. 60.5%, *p* = 0.024). Almost one-third of the patients suffered from GI complications, which were more frequent in those patients needing PN, with significant differences in elevated gastric residual volume and mesenteric ischemia (Table 1). In relation to outcomes, more days on mechanical ventilation, more need of RRT, longer ICU stay and a trend toward lower mortality were found in patients treated with PN (Table 1). Also, patients who need PN required RRT more frequently (HR:1.316, 95% CI:1.160–1.626, *p* = 0.001) and experienced a higher mortality during ICU admission (HR:1.460, 95% CI:0.22–0.954, *p* = 0.037). Differences in 28-day mortality according to the pattern of feeding (as shown in Figure 2) were not observed (*p* = 0.144).

**Figure 4:**
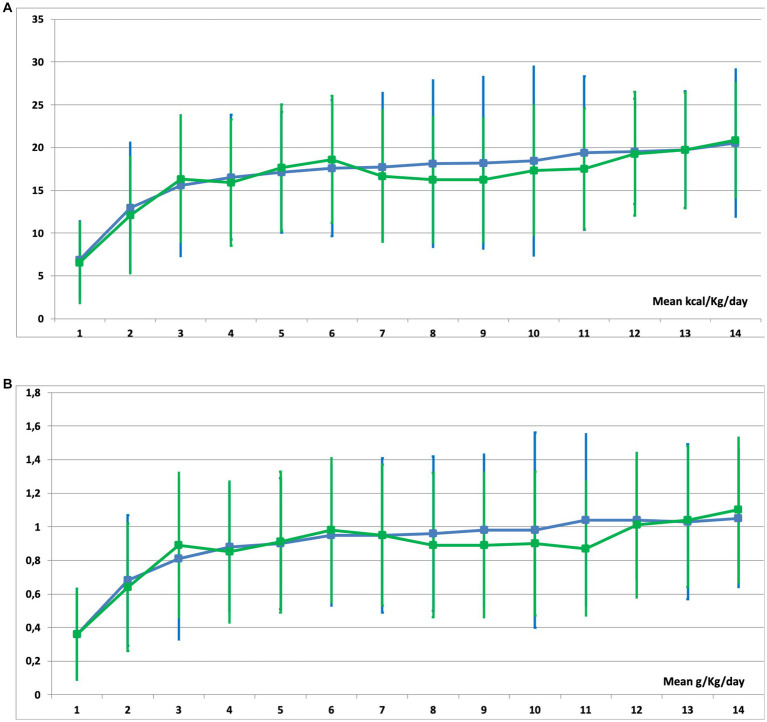
Mean caloric **(A)** and protein **(B)** delivery in patients receiving enteral nutrition (EN) (green line) and those receiving EN with parenteral nutrition (PN) (blue line) during their stay in the ICU.

Even though patients who needed EN exclusively performed better during early nutritional therapy, there were no statistical differences in mean energy (Figure 4A) and protein (Figure 4B) delivery as compared to the EN-PN group (Table 2). In relation to laboratory analyses, almost all patients presented some type of electrolyte disbalance during ICU admission, with abnormalities in lipid profile (especially triglycerides), lower levels of blood proteins (albumin and prealbumin), and worst parameters of renal function (urea and creatinine) among patients needing PN. Laboratory values with statistically significant differences between the groups of EN and EN-PN are shown in Table 3. Patients did not develop significant increase in liver enzymes and bilirubin during PN administration. The complete list of laboratory results is included in the Supplementary Table S2.

**Table 2:** Mean caloric and protein requirements in ICU patients receiving enteral nutrition (EN) and enteral nutrition with parenteral nutrition (EN-PN) during ICU stay.

Days of ICU stay	Kcal/kg/day, mean ± SD				Proteins, g/kg/day, mean ± SD			
	All patients (*n* = 443)	EN (*n* = 400)	EN-PN (*n* = 43)	*P* value	All patients (*n* = 443)	EN (*n* = 400)	EN-PN (*n* = 43)	*P* value
1	6.83 ± 4.71	6.86 ± 4.73	6.52 ± 4.55	*0.65*	0.36 ± 0.27	0.36 ± 0.27	0.36 ± 0.27	*0.91*
2	12.88 ± 6.88	12.97 ± 6.81	12.07 ± 7.56	*0.42*	0.68 ± 0.38	0.68 ± 0.38	0.64 ± 0.39	*0.45*
3	15.63 ± 15.44	15.56 ± 7.36	16.31 ± 8.23	*0.53*	0.82 ± 0.43	0.81 ± 0.43	0.89 ± 0.48	*0.30*
4	16.47 ± 7.37	16.54 ± 7.38	15.89 ± 7.28	*0.59*	0.87 ± 0.41	0.88 ± 0.42	0.85 ± 0.38	*0.76*
5	17.17 ± 7.35	17.11 ± 7.40	17.65 ± 7.08	*0.66*	0.91 ± 0.42	0.90 ± 0.42	0.91 ± 0.39	*0.87*
6	17.73 ± 7.51	17.61 ± 7.45	18.61 ± 7.98	*0.44*	0.95 ± 0.43	0.95 ± 0.43	0.98 ± 0.42	*0.63*
7	17.60 ± 7.81	17.72 ± 7.71	16.66 ± 8.66	*0.46*	0.95 ± 0.43	0.95 ± 0.42	0.95 ± 0.46	*0.98*
Mean 1st week	10.16 ± 4.44	10.11 ± 4.37	10.62 ± 5.09	*0.47*	0.73 ± 0.33	0.72 ± 0.33	0.79 ± 0.27	*0.23*
8	17.94 ± 7.62	18.13 ± 7.33	16.27 ± 9.74	*0.22*	0.95 ± 0.43	0.96 ± 0.43	0.89 ± 0.46	*0.40*
9	17.97 ± 7.61	18.20 ± 7.24	16.22 ± 10.03	*0.20*	0.97 ± 0.43	0.98 ± 0.43	0.89 ± 0.45	*0.34*
10	18.31 ± 8.00	18.44 ± 7.56	17.32 ± 11.04	*0.51*	0.98 ± 0.45	0.98 ± 0.43	0.90 ± 0.58	*0.36*
11	19.13 ± 7.32	19.36 ± 7.07	17.50 ± 8.95	*0.24*	1.02 ± 0.42	1.04 ± 0.40	0.87 ± 0.51	*0.06*
12	19.50 ± 7.12	19.53 ± 7.25	19.28 ± 6.15	*0.88*	1.03 ± 0.42	1.04 ± 0.43	1.01 ± 0.35	*0.79*
13	19.74 ± 6.75	19.75 ± 6.76	19.69 ± 6.82	*0.97*	1.03 ± 0.45	1.03 ± 0.44	1.04 ± 0.46	*0.93*
14	20.53 ± 6.83	20.50 ± 6.67	20.87 ± 8.60	*0.87*	1.06 ± 0.43	1.05 ± 0.43	1.10 ± 0.41	*0.71*
Mean 2nd week	14.59 ± 5.58	14.50 ± 5.60	15.46 ± 5.31	*0.28*	0.77 ± 0.34	0.76 ± 0.34	0.83 ± 0.28	*0.24*

*P* significant value is written in bold.

**Table 3:** Significant differences in laboratory values between receiving enteral nutrition (EN) and enteral nutrition with parenteral nutrition (EN-PN) during ICU stay.

	All patients (*n* = 443)	EN (*n* = 400)	EN-PN (*n* = 43)	*P* value
Lipid profile				
Triglycerides, mg·dL^−1^				
On ICU admission	139.00 ± 94.87	130.58 ± 71.95	210.20 ± 192.48	***<0.001***
On day 3	160.33 ± 101.11	151.83 ± 91.21	223.55 ± 143.74	***0.001***
On day 7	173.93 ± 114.00	166.07 ± 85.34	226.94 ± 168.83	***0.03***
On ICU discharge	165.43 ± 91.27	157.79 ± 85.34	218.56 ± 113.64	***0.003***
Hypertriglyceridemia (>350 mg·dL^−1^)	28 (6.3%)	19 (4.7%)	9 (20.9%)	***0.003***
Cholesterol on day 7, mg·dL^−1^	136.03 ± 42.06	140.92 ± 41.02	101.83 ± 33.09	***<0.001***
Hypercholesterolemia (>240 mg·dL^−1^)	16 (3.6%)	13 (3.2%)	3 (7.0%)	***0.04***
Low HDL-C (<40 mg·dL^−1^)	176 (39.7%)	158 (39.5%)	18 (41.9%)	***0.03***
Liven function tests				
AST/GOT on day 3, IU·L^−1^	127.00 ± 532.03	105.47 ± 423.85	334.05 ± 1129.18	***0.04***
AST/GOT on ICU discharge, IU·L^−1^	72.80 ± 381.57	51.64 ± 93.39	258.83 ± 1162.99	***0.005***
Bilirubin on ICU discharge, mg·dL^−1^	0.80 ± 1.18	0.74 ± 1.10	1.34 ± 1.70	***0.008***
Alkaline phosphatase on day 3, IU·L^−1^	96.86 ± 67.09	93.91 ± 63.34	126.62 ± 93.83	***0.02***
Blood cell count				
Leukocytosis (>11·10^9^·L^−1^)	345 (77.9%)	310 (77.5%)	35 (81.4%)	***0.006***
Platelets on day 7, cells·10^9^·L^−1^	228.87 ± 120.95	235.80 ± 121.75	173.58 ± 99.57	***0.003***
Renal function tests				
Creatinine, mg·dL^−1^				
On ICU admission	1.42 ± 1.28	1.37 ± 1.29	1.83 ± 1.14	***0.03***
On ICU discharge	0.96 ± 1.01	0.92 ± 0.99	1.26 ± 1.14	***0.04***
Renal failure, creatinine >1.3 mg·dL^−1^	252 (56.9%)	220 (55.0%)	32 (74.4%)	***0.04***
Proteins				
Prealbumin, mg·L^−1^				
On day 7	201.24 ± 106.83	209.82 ± 106.66	133.67 ± 83.49	***0.007***
On ICU discharge	206.38 ± 92.14	211.76 ± 93.01	160.13 ± 71.06	***0.04***
Low prealbumin levels, <200 mg·L^−1^	230 (51.9%)	206 (51.5%)	24 (55.8%)	***0.03***
Albumin, mg·L^−1^				
On ICU admission	3.07 ± 0.65	3.10 ± 0.62	2.71 ± 0.79	***0.001***
On day 3	2.80 ± 0.56	2.84 ± 0.56	2.44 ± 0.48	***<0.001***
On day 7	2.75 ± 0.58	2.79 ± 0.57	2.33 ± 0.42	***<0.001***
On ICU discharge	2.93 ± 0.62	2.98 ± 0.60	2.55 ± 0.64	***<0.001***
Low albumin levels, <30 mg·L^−1^	421 (95.0%)	37 (94.7%)	42 (97.8%)	***<0.001***
C-reactive protein on ICU discharge, mg·L^−1^	69.59 ± 83.89	64.11 ± 77.26	116.84 ± 119.17	***0.001***

ICU, intensive care unit; HDL-C, high-density lipoprotein cholesterol; AST, aspartate aminotransferase; GOT, glutamic-oxaloacetic transaminase. Data as mean ± standard deviation or frequencies and percentages in parenthesis. *P* significant value is written in bold.

Once adjusted by confounding factors, the multivariate analysis showed that initial higher SOFA score and serum triglycerides on ICU admission were independently associated with the need of PN after initiation of EN, whereas higher serum albumin levels on ICU admission were inversely associated with the need of PN (Table 4).

**Table 4:** Results of multivariate analysis of factors associated with the need of parenteral nutrition (PN) in patients receiving enteral nutrition (EN) during ICU stay.

Variables on ICU admission	Hazard ratio (95% confidence interval)	*P* value
SOFA	1.221 (1.057–1.410)	***0.007***
Serum triglycerides, mg·dL^−1^	1.004 (1.001–1.007)	***0.003***
Serum albumin, g·L^−1^	0.424 (0.210–0.687)	***0.016***

Model adjusted by age, type of patient, severity of illness, and length of nutritional therapy. SOFA, Sequential Organ Failure Assessment. *P* significant value is written in bold.

## 4. Discussion

This multicenter study shows the higher need of PN associated with an initial higher organ failure (i.e., higher SOFA score) on ICU admission in those patients in whom EN had been chosen as initial route of nutrition therapy in ICU patients. We have also shown the association of nutrition-related laboratory parameters, such as triglyceride and albumin levels, with this phenomenon. This is of special importance since there are not validated tools to assess and predict the lack of efficacy of EN to provide adequate nutrition therapy during ICU stay (29). Despite this study was observational, it was performed by participating investigators interested in nutrition, and we may assume that the indication of PN was not related to barrier factors influencing enteral feeding (e.g., delay in the start of EN) but with difficulties in achieving sufficient nutritional requirements based on the clinical condition of the patient (e.g., severity of critical illness and GI complications). The novelty of this research also remains in the analysis of objective and measurable variables, and the combination of all these observations in a single multivariable analysis, which may add new knowledge related to the quality of in-data in observational studies. Finally, we have described the characteristics of EN in a large ICU population, examining differences in clinical features, energy and protein intake, GI complications, and outcome between EN and EN-PN subgroups.

In our cohort, we showed that patients with a SOFA score > 6 on ICU admission were more likely to need PN after initiation of EN. The association of higher SOFA score on ICU admission with higher need of PN during ICU stay is probably related to GI dysfunction. GI dysfunction, which is defined as a transient state (e.g., gastroparesis or ileus) secondary to or in the context of severe illness (i.e., major surgery, sepsis, malnutrition, etc.) has been recognized as a main cause for the use of PN in ICU patients (24). Moreover, impaired gastric motility in critically ill patients is associated with an increased gastric bacterial colonization, pulmonary aspiration and progressive malnutrition leading to adverse outcomes. This is reflected by higher rates of GI complications, which were more frequent in the EN-PN subgroup in our population (27–29). This is not surprising since some of the complications (e.g., high gastric residual volume) are related with the inability of providing adequate nutrition delivery and the need of PN.

It is estimated that at least 60% of ICU patients are affected by some form of GI dysfunction, and that in 30% of patients in whom EN is attempted the feeding route needs to be modified to PN route because of not achieving prescribed nutrient provision or the occurrence of GI complications or both (25). In our study, the use of PN was related to paralytic ileus or the inability to fully tolerate EN, accounted for the impossibility of achieving adequate and sufficient nutritional requirements with EN alone in 10% of the patients. The reasons behind this lower incidence may be probably related with a little presence of barrier factors influencing enteral feeding based on the nature of participating sites. In addition, we did not find differences regarding caloric and protein delivery when EN and EN-PN subgroups where compared. Thus, our findings may also reflect the importance of PN for optimal nutrition delivery in those patients with initial higher disease severity.

Nutrition-related laboratory parameters, such as higher triglycerides on ICU admission, were associated with the need of PN in patients receiving EN, whereas high albumin levels appeared to be a protective factor. Hypertriglyceridemia has been reported to be associated with high-grade inflammation increasing the risk of different conditions, such as acute pancreatitis (30). Indeed, elevated triglyceride concentrations have been reported in patients with systemic inflammatory response (i.e., stress response) due to sepsis or other diseases (31). Hypoalbuminemia, one of the most prevalent abnormalities in ICU patients, is associated with inflammation in a part as a reflection of the extent of physiologic stress resulting from critical illness (32, 33). During critical illness hepatic reprioritization of protein synthesis and an increase in capillary permeability occurs, resulting in lower albumin production and redistribution of serum proteins, respectively (34). Hypertriglyceridemia and hypoalbuminemia, together with C-reactive protein, are probably metabolic factors associated to systemic inflammation and not only to poor or altered metabolic and nutritional status. The association between systemic inflammation and malnutrition is closely linked with the occurrence of GI failure, and it may help to explain our finding regarding nutrition-related laboratory markers (34).

Hypertriglyceridemia is related with metabolic response during acute phase (i.e., first 48–72 h of ICU admission) since inflammatory mediators (i.e., hormones, cytokines, lymphokines) released during the stress response stimulates lipolysis (5). Lipolysis triggers hydrolysis of triglycerides in adipose tissue, producing fatty acids and glycerol. Glycerol is used for gluconeogenesis in the liver, contributing to glucose production, and fatty acids are used by the liver and muscles, converted to ketone bodies, or re-esterified (6). However, lipid metabolism, and more specifically lipid oxidation, is limited due to the lower availability of oxygen during acute phase (i.e., tissular hypoxia), which is also related with the occurrence of mitochondrial dysfunction during critical illness (7, 8). In addition, a higher delivery of lipid metabolites is not associated with an effective inhibition of lipolysis (similar to other metabolic pathways), resulting in an even higher concentration of lipid metabolites when patients received nutrition therapy and some lipid drugs (e.g., propofol) (7). The rate of fatty acid released may also exceed energy needs, and those that are not oxidized may be re-esterified to triglyceride (6). Thus, the occurrence of hypertriglyceridemia may be related with the degree of stress response in critically ill on the basis of an ineffective lipid metabolism.

The nutritional status of ICU patients deteriorates rapidly after admission due to severe catabolism caused by proinflammatory cytokines and hormones, even when patients are well nourished (35, 36). Also, half of the patients showed high nutritional risk (i.e., mNUTRIC score ≥ 5) in our population and in the univariate analysis, high nutritional risk was associated with the need of PN. However, this may be more linked with disease severity rather than the relationship of higher nutritional risk with GI dysfunction, since mNUTRIC score includes ICU prognosis scores, such as SOFA and APACHE II.

The duration of mechanical ventilation, the need of RRT, and the length of stay in the ICU were higher in the EN-PN subgroup, as well as a trend toward higher 28-day mortality. Despite PN avoids caloric debt and it may improve outcome of patients who do not tolerate fully EN, the benefit over mortality is not entirely elucidated (26, 37). This trend toward higher mortality in the EN-PN subgroup may reflect the severity disease of those patients, which also may be a feature of additional organ dysfunction, such as GI dysfunction, more than a lack of benefit of PN *per se*. As we underlined above, we showed no difference in terms of caloric or protein delivery between EN and EN-PN subgroups of patients, which may reflect the adequacy of PN in providing appropriate nutrition therapy when EN is not completely or partially feasible.

**Figure 5:**
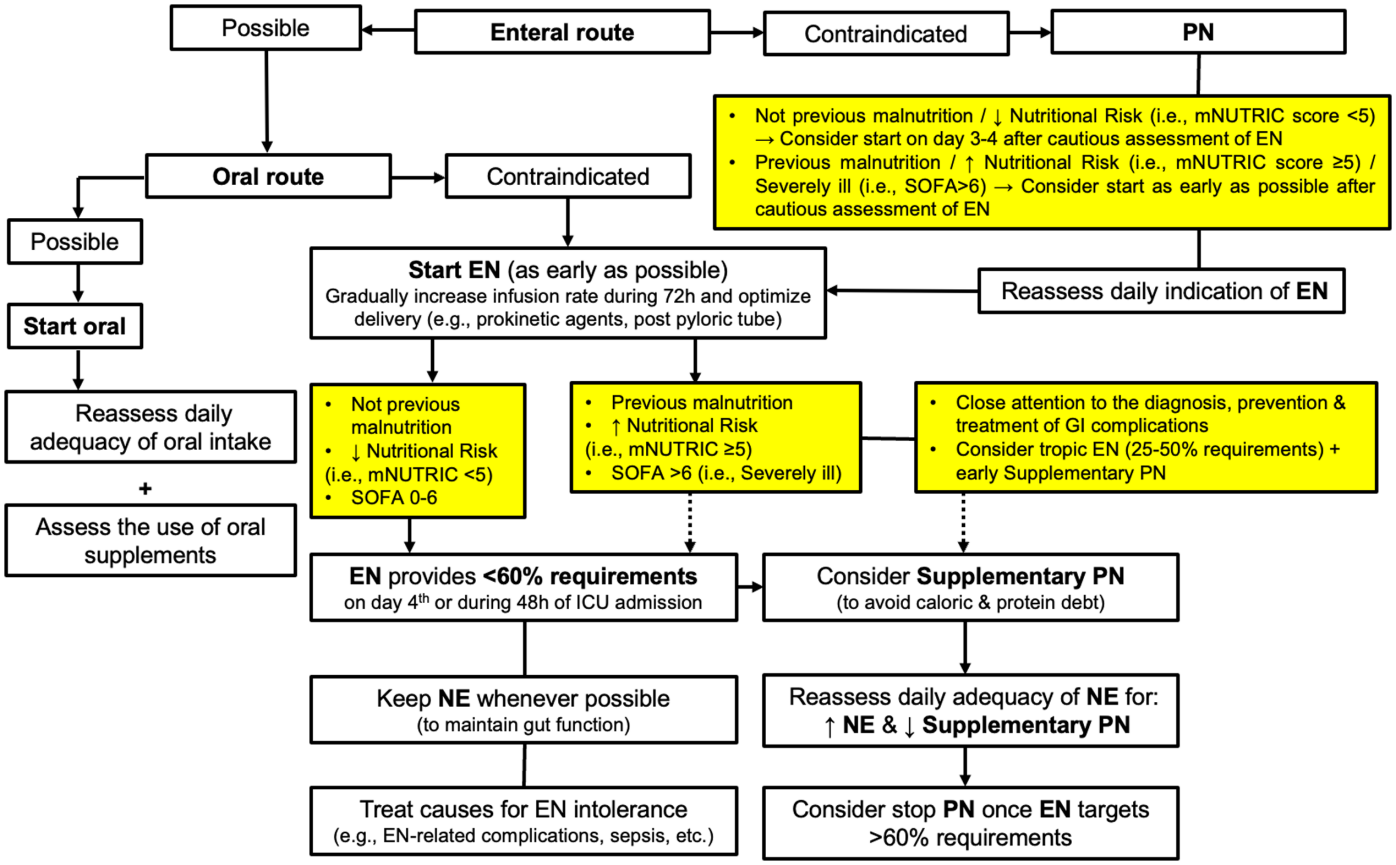
Proposal of a modified algorithm to select the feeding route for giving nutrition therapy in ICU patients. EN, Enteral Nutrition; PN, Parenteral Nutrition; mNUTRIC, modified Nutrition Risk in the Critically II score; SOFA, Sequential Organ Failure Assessment score.

Even though our results are debatable, they are clinically relevant to provide a basis for future trials, especially regarding the strategy in giving nutrition therapy in the most critically ill with high organ failure on admission or during ICU stay. It is important to anticipate and identify ICU patients who are not able to tolerate adequate EN for supplying energy and protein needs, and those would benefit from supplemental PN (38, 39). We have hypothesized that those patients needing PN may benefit from a different nutritional strategy, such as the early use of trophic nutrition together with supplemental PN, in order to avoid the deleterious effects of undernutrition, especially in patients with established malnutrition or at high nutritional risk. This would minimize the risks that GI or EN-related complications may entail for severe critically ill (e.g., invasive ventilated patients with shock) (40). On the basis of our findings and the present discussion, we would like to suggest a proposal of a modified algorithm to select the feeding route for giving nutrition therapy (Figure 5) (13, 38, 39).

### 4.1. Limitations and strengths

Limitations of the study are mainly related to the observational nature, which only allows to assess associations, the heterogeneity of participants, and the low number of patients in the EN-PN group (*n* = 43). The latter did not allow subgroup analyses based on the type of patient (i.e., medical, surgical and trauma). However, these limitations have been counterbalanced by means of statistical analysis that have minimized the impact of confounders (e.g., confounding by indication), as well as their influence in our results (described in the Methods section). Strengths of the study also include the large sample size (*n* = 443), the prospective design with a high data quality, the participation of a high number of ICUs, and the follow-up of nutrition therapy during 14 days after ICU admission, as well as the fact that data here reported reflect real-world practice of intensivists delivering nutrition therapy (16).

## 5. Conclusion

In this study, patients admitted to the ICU who need EN may be frequently at nutritional risk and PN may provide adequate delivery of nutrition therapy when needed. A higher organ failure (i.e., higher SOFA) and nutrition-related laboratory parameters, such as albumin and triglycerides, on ICU admission may be associated with the need of PN after starting EN therapy. This may be related with a higher occurrence of GI complications, a feature of GI dysfunction.

## Data availability statement

The raw data supporting the conclusions of this article will be made available by the authors, without undue reservation.

## Ethics statement

The studies involving humans were approved by Clinical Research Ethics Committee of Hospital Universitari de Bellvitge (Barcelona, Spain). The studies were conducted in accordance with the local legislation and institutional requirements. The ethics committee/institutional review board waived the requirement of written informed consent for participation from the participants or the participants’ legal guardians/next of kin because observational nature of the study & Compliance with legal requirements.

## Author contributions

JL-D, TG-C, and LS-G conceived, designed, and coordinated the study. TG-C, JL-D, and JM performed the statistical analysis and developed the first draft of the manuscript. All authors collected the data during the study period and participated in the revision, involved in the interpretation of the results, critically revised the manuscript, and approved the final version.

## Funding

This study was supported and received a grant from the Spanish Society of Nutrition and Metabolism (SENPE).

## Conflict of interest

The authors declare that the research was conducted in the absence of any commercial or financial relationships that could be construed as a potential conflict of interest.

## Publisher’s note

All claims expressed in this article are solely those of the authors and do not necessarily represent those of their affiliated organizations, or those of the publisher, the editors and the reviewers. Any product that may be evaluated in this article, or claim that may be made by its manufacturer, is not guaranteed or endorsed by the publisher.

## Abbreviations

ICU: Intensive Care Unit
EN: Enteral Nutrition
PN: Parenteral Nutrition
GI: Gastrointestinal
APACHE: Acute Physiology and Chronic Health Evaluation
SAPS: Simplified Acute Physiology Score
SOFA: Sequential Organ Failure Assessment
mNUTRIC: modified Nutrition Risk in the Critically II
RRT: Renal Replacement Therapy
