# Supplementary material for: Factors associated with the need of parenteral nutrition in critically ill patients after the initiation of enteral nutrition therapy
Source: Front Nutr. 2023 Aug 24;10:1250305. doi: 10.3389/fnut.2023.1250305 (PMC10491892; doi:10.3389/fnut.2023.1250305)
Supplement: Supplementary file 1 [file Data_Sheet_1.docx]

**Table S1.** Number and location of participating hospitals throughout Spain.

|  | Autonomous Community | Hospital (City) |
| --- | --- | --- |
|  | Catalonia (Catalunya) | Hospital Universitari Arnau de Vilanova (Lleida) |
|  |  | Hospital Universitari Josep Trueta (Girona) |
|  |  | Hospital Universitari Bellvitge (L’Hospitalet del Llobregat, Barcelona) |
|  |  | Hospital General de Granollers (Granollers, Barcelona) |
|  |  | Hospital de la Santa Creu i Sant Pau (Barcelona) |
|  |  | Hospital Universitario Germans Trias i Pujol (Badalona; Barcelona) |
|  |  | Hospital del Mar (Barcelona) |
|  |  | Hospital Plató (Barcelona) |
|  |  | Hospital Mutua Terrasa (Terrasa; Barcelona) |
|  |  | Hospital de Mataró (Mataró; Barcelona) |
|  | Comunidad de Madrid | Hospital Universitario 12 de octubre (Madrid) |
|  |  | Hospital Universitario Infanta Sofía (San Sebastián de los Reyes, Madrid) |
|  |  | Hospital Universitario de Getafe (Getafe; Madrid) |
|  |  | Hospital Infanta Cristina (Parla; Madrid) |
|  |  | Hospital Príncipe de Asturias (Alcalá de Henares; Madrid) |
|  |  | Hospital Universitario Severo Ochoa (Leganés; Madrid) |
|  |  | Hospital Universitario de Fuenlabrada (Madrid) |
|  | Comunitat Valenciana | Hospital Sant Joan d’ Alacant (Alicante) |
|  |  | Hospital Universitari Doctor Peset (Valencia) |
|  |  | Hospital General Universitario de Alicante (Alicante) |
|  |  | Hospital General Universitario de Castellón (Castelló de la Plana) |
|  |  | Hospital Clínico Universitario de Valencia (Valencia) |
|  | Navarra | Complejo Hospitalario de Navarra (Pamplona) |
|  | Balearic Islands | Hospital de Manacor (Manacor; Mallorca) |
|  | Aragón | Hospital San Jorge (Huesca) |
|  |  | Hospital Royo Villanova (Zaragoza) |
|  |  | Hospital Clínico Lozano Blesa (Zaragoza) |
|  |  | Hospital Miguel Servet (Zaragoza) |
|  |  | Hospital de Barbastro (Barbastro; Huesca) |
|  | Andalucía | Hospital Regional Universitario Carlos Haya (Málaga) |
|  |  | Hospital Universitario de Puerto Real (Puerto Real; Cádiz) |
|  | Murcia | Hospital General Universitario Reina Sofia (Murcia) |
|  | Castilla-León | Hospital Río Hortega (Valladolid) |
|  |  | Hospital Clínico Universitario de Valladolid (Valladolid) |
|  |  | Hospital Universitario de Burgos (Burgos) |
|  |  | Hospital Virgen de la Concha (Zamora) |
|  | Galicia | Hospital Álvaro Cunqueiro (Vigo; Pontevedra) |
|  |  | Hospital Universitario de A Coruña (La Coruña) |

**Table S2.** Laboratory abnormalities of the patients receiving enteral nutrition admitted in the ICU: electrolytes **(A)**, lipid profile **(B)**, liver parameters **(C)**, and haematology, renal and blood proteins **(D)**.

| **A** | All patients  (n=443) | EN  (n=400) | EN with PN  (n=43) | *P* |
| --- | --- | --- | --- | --- |
| ***Electrolytes*** | | | | |
| Any type of electrolyte disbalance | 98.42 % (436) | 98.25 % (393) | 100 % (43) | *0.35* |
| Na^+^ (on admission) | 140.11 ± 5.99 \| 140 (109-168) | 140.08 ± 5.80 \| 140 (109-168) | 140.39 ± 7.63 \| 140 (125-159) | *0.74* |
| Na^+^ (day 3) | 142.62 ± 5.23 \| 142 (128-168) | 142.71 ± 5.21 \| 142.90 (130-168) | 141.74 ± 5.46 \| 141 (128-155) | *0.25* |
| Na^+^ (day 7) | 142.91 ± 6.02 \| 142 (127-168) | 142.84 ± 5.94 \| 142 (127-168) | 143.41 ± 6.68 \| 143 (132-161) | *0.59* |
| Na^+^ (on discharge) | 139.98 ± 7.52 \| 140 (63-163) | 140.15 ± 7.67 \| 140 (63-168) | 138.37 ± 5.75 \| 138 (126-151) | *0.14* |
| Hyponatremia (Na^+^ < 135 mmol·L^-1^) | 25.28 % (112) | 23.25 % (93) | 44.19 % (19) | *0.40* |
| Hypernatremia (Na^+^ > 145 mmol·L^-1^) | 51.92 % (230) | 50.75 % (203) | 62.79 % (27) | *0.98* |
| K^+^ (on admission) | 4.11 ± 0.69 \| 4.00 (2.60-6.90) | 4.10 ± 0.66 \| 4.00 (2.60-6.90) | 4.22 ± 0.87 \| 4.05 (2.98-6.90) | *0.29* |
| K^+^ (day 3) | 3.96 ± 0.53 \| 3.90 (1.88-5.70) | 3.96 ± 0.54 \| 3.90 (1.88-5.70) | 3.97 ± 0.53 \| 3.81 (3.08-5.40) | *0.86* |
| K^+^ (day 7) | 4.17 ± 0.64 \| 4.10 (2.51-6.60) | 4.19 ± 0.62 \| 4.10 (2.68-6.60) | 3.98 ± 0.74 \| 3.83 (2.51-5.70) | *0.06* |
| K^+^ (on discharge) | 4.14 ± 0.67 \| 4.10 (2.50-7.40) | 4.13 ± 0.65 \| 4.10 (2.50-7.10) | 4.29 ± 0.82 \| 4.16 (3.05-7.40) | *0.13* |
| Hypokalaemia (K^+^< 3.5 mmol·L^-1^) | 42.21 % (187) | 41.00 % (164) | 53.49 % (23) | *0.38* |
| Hyperkalaemia (>5 mmol·L^-1^) | 20.99 % (93) | 20.00 % (80) | 30.23 % (13) | *0.77* |
| Mg^2+^ (on admission) | 0.88 ± 0.31 \| 0.82 (0.38-3.50) | 0.87 ± 0.31 \| 0.81 (0.38-3.50) | 0.96 ± 0.33 \| 0.95 (0.47-2.24) | *0.14* |
| Mg^2+^ (day 3) | 0.94 ± 0.28 \| 0.86 (0.43-2.40) | 0.94 ± 0.28 \| 0.87 (0.43-2.40) | 0.92 ± 0.28 \| 0.84 (0.58-2.10) | *0.78* |
| Mg^2+^ (day 7) | 0.93 ± 0.24 \| 0.90 (0.47-2.22) | 0.93 ± 0.22 \| 0.90 (0.47-2.22) | 0.94 ± 0.38 \| 0.82 (0.53-2.20) | *0.81* |
| Mg^2+^ (on discharge) | 0.88 ± 0.23 \| 0.84 (0.45-2.30) | 0.87 ± 0.22 \| 0.82 (0.45-2.30) | 0.95 ± 0.28 \| 0.86 (0.66-1.90) | *0.09* |
| Hypomagnesaemia (Mg^2+^<0.74 mmol·L^-1^ /<1.8 mg·dL^-1^) | 29.57 % (131) | 29.50 % (118) | 30.23 % (13) | *0.66* |
| Hypermagnesaemia (> 2.6 mg/dL / > 1.05 mmol/L) | 25.51 % (113) | 25.25 % (101) | 27.91 % (12) | *0.40* |
| Phosphate (on admission) | 1.16 ± 0.58 \| 1.00 (0.20-4.10) | 1.14 ± 0.57 \| 1.00 (0.20-4.10) | 1.30 ± 0.65 \| 1.10 (0.50-3.10) | *0.17* |
| Phosphate (day 3) | 1.09 ± 0.58 \| 0.96 (0.16-5.10) | 1.09 ± 0.58 \| 0.96 (0.16-5.10) | 1.11 ± 0.59 \| 0.96 (0.26-2.70) | *0.84* |
| Phosphate (day 7) | 1.17 ± 0.49 \| 1.09 (0.38-3.70) | 1.18 ± 0.50 \| 1.09 (0.38-3.70) | 1.12 ± 0.46 \| 1.12 (0.39-1.97) | *0.58* |
| Phosphate (on discharge) | 1.17 ± 0.46 \| 1.10 (0.39-3.90) | 1.15 ± 0.42 \| 1.10 (0.39-3.30) | 1.30 ± 0.71 \| 1.05 (0.39-3.90) | *0.10* |
| Hypophosphatemia (Phosphate<0.87mmol·L^-1^ /<2.7mg·dL^-1^) | 43.12 % (191) | 42.75 % (171) | 46.51 % (20) | *0.14* |
| Hyperphosphatemia (> 4.5 mg/dL / >1.46 mmol/L) | 25.06 % (111) | 23.75 % (95) | 37.21 % (16) | *0.91* |
| Ca^2+^ (on admission) | 2.07 ± 0.20 \| 2.07 (1.52-2.80) | 2.08 ± 0.20 \| 2.08 (1.52-2.80) | 2.00 ± 0.22 \| 2.01 (1.52-2.49) | ***0.04*** |
| Ca^2+^ (day 3) | 2.09 ± 0.20 \| 2.07 (1.55-2.95) | 2.09 ± 0.20 \| 2.07 (1.55-2.95) | 2.10 ± 0.21 \| 2.12 (1.65-2.47) | *0.77* |
| Ca^2+^ (day 7) | 2.12 ± 0.18 \| 2.10 (1.52-2.61) | 2.12 ± 0.18 \| 2.12 (1.57-2.61) | 2.04 ± 0.23 \| 2.02 (1.52-2.45) | ***0.04*** |
| Ca^2+^ (on discharge) | 2.04 ± 0.36 \| 2.12 (0.99-2.80) | 2.04 ± 0.36 \| 2.11 (0.99-2.80) | 2.00 ± 0.39 \| 2.12 (1.13-2.49) | *0.57* |
| Hypocalcaemia (Ca^2+^<2.13 mmol·L^-1^/ <8.6 mg·dL^-1^) | 77.88 % (345) | 77.25 % (309) | 83.72 % (36) | *0.56* |
| Hypercalcaemia (>10.7 mg/dL / 2.6 mmol/L) | 2.48 % (11) | 2.75 % (11) | 0 % (0) | *1* |

Na^+^: Sodium plasma levels; K^+^: Potassium plasma levels; Mg^2+^: Magnesium plasma levels; Ca^2+^: Calcium plasma levels.

| **B** | All patients  (n=443) | EN  (n=400) | EN with PN  (n=43) | *P* |
| --- | --- | --- | --- | --- |
| ***Lipid profile*** | | | | |
| Triglycerides (on admission) | 139.00 ± 94.87 \| 119 (31-810) | 130.58 ± 71.95 \| 119 (31-558) | 210.20 ± 192.48 \| 124.50 (59-810) | ***<0.001*** |
| Triglycerides (day 3) | 160.33 ± 101.11 \| 130 (11-739) | 151.83 ± 91.21 \| 129 (11-739) | 223.55 ± 143.74 \| 151 (88-477) | ***0.001*** |
| Triglycerides (day 7) | 173.93 ± 114.00 \| 146 (30-743) | 166.07 ± 85.34 \| 144 (30-575) | 226.94 ± 168.83 \| 164 (74-743) | ***0.03*** |
| Triglycerides (on discharge) | 165.43 ± 91.27 \| 148 (35-604) | 157.79 ± 85.34 \| 137 (35-604) | 218.56 ± 113.64 \| 203 (67.40-477) | ***0.003*** |
| Hypertriglyceridemia (>350mg·dL^-1^) | 6.32 % (28) | 4.75 % (19) | 20.93 % (9) | ***0.003*** |
| Cholesterol (on admission) | 130.94 ± 46.19 \| 126 (33-335) | 131.66 ± 42.13 \| 128 (33-324) | 125.04 ± 72.37 \| 100 (45-335) | *0.50* |
| Cholesterol (day 3) | 127.05 ± 42.06 \| 121 (29-298) | 128.75 ± 40.63 \| 122.30 (29-253) | 112.10 ± 51.77 \| 103.50 (40-298) | *0.09* |
| Cholesterol (day 7) | 136.03 ± 42.06 \| 134 (29-239) | 140.92 ± 41.02 \| 140 (29-239) | 101.83 ± 33.09 \| 101 (52.10-157) | ***<0.001*** |
| Cholesterol (on discharge) | 150.97 ± 51.58 \| 145 (36-335) | 152.99 ± 49.42 \| 147.31 (36-293) | 134.35 ± 66.06 \| 123 (52.10-335) | *0.14* |
| High cholesterol levels (>5.2 mmol·L^-1^/ >200 mg·dL^-1^) | 10.84 % (48) | 11.00 % (44) | 9.30 % (4) | ***0.004*** |
| High cholesterol levels (>6.2 mmol·L^-1^/ >240 mg·dL^-1^) | 3.61 % (16) | 3.25 % (13) | 6.97 % (3) | ***0.04*** |
| HDL (on admission) | 37.91 ± 19.59 \| 36 (3.10-135) | 37.84 ± 17.47 \| 36 (3.10-86) | 38.49 ± 33.22 \| 32.30 (7-135) | *0.90* |
| HDL (day 3) | 60.50 ± 94.63 \| 31 (5-484) | 63.46 ± 98.40 \| 32 (5-484) | 32.00 ± 35.05 \| 27 (5-141) | *0.25* |
| HDL (day 7) | 32.42 ± 21.23 \| 27 (4-117) | 32.30 ± 18.68 \| 28.10 (4-113) | 33.31 ± 36.00 \| 18 (7-117) | *0.87* |
| HDL (on discharge) | 32.50 ± 15.95 \| 30.15 (7.50-120) | 32.96 ± 15.28 \| 31 (7.50-120) | 28.38 ± 21.49 \| 20 (8-85) | *0.37* |
| Low HDL levels (<1mmol·L^-1^/ <40 mg·dL^-1^) | 39.73 % (176) | 39.50 % (158) | 41.86 % (18) | ***0.03*** |
| LDL (on admission) | 65.38 ± 31.18 \| 66 (7.60-171) | 67.05 ± 30.98 \| 67 (7.60-171) | 50.68 ± 30.08 \| 49 (15-127.60) | *0.05* |
| LDL (day 3) | 176.90 ± 382.23 \| 71 (8-2988) | 185.99 ± 398.41 \| 71.50 (8-2988) | 84.41 ± 101.12 \| 55.50 (15-395) | *0.38* |
| LDL (day 7) | 80.38 ± 46.07 \| 75.40 (10-334) | 82.02 ± 46.34 \| 77.50 (10-334) | 69.31 ± 44.31 \| 59 (21-181) | *0.36* |
| LDL (on discharge) | 87.33 ± 40.70 \| 81.50 (12-209) | 89.26 ± 39.76 \| 82.02 (12-209) | 69.96 ± 46.83 \| 60 (13.80-174) | *0.14* |
| High LDL levels (>4.1mmol·L^-1^/ >160 mg·dL^-1^) | 6.77 % (30) | 6.75 % (27) | 6.97 % (3) | *0.32* |
| High LDL levels (>4.9mmol·L^-1^/ >190 mg·dL^-1^) | 5.19 % (23) | 5.50 % (22) | 2.33 % (1) | *0.62* |

HDL: High-density lipoprotein cholesterol; LDL: low-density lipoprotein colesterol.

| **C** | All patients  (n=443) | EN  (n=400) | EN with PN  (n=43) | *P* |
| --- | --- | --- | --- | --- |
| ***Liver parameters*** | | | | |
| ALT/ GPT (on admission) | 75.33 ± 182.67 \| 27 (5-1994) | 69.75 ± 161.76 \| 27 (5-1994) | 128.19 ± 318.03 \| 29 (9-1652) | *0.06* |
| ALT/ GPT (day 3) | 100.89 ± 364.09 \| 27 (4-3674) | 90.24 ± 330.68 \| 27 (5-3674) | 203.12 ± 595.76 \| 32 (8-2556) | *0.10* |
| ALT/ GPT (day 7) | 79.29 ± 263.10 \| 40 (4-4059) | 80.14 ± 277.65 \| 41.50 (4-4059) | 73.06 ± 109.21 \| 33 (9-427.80) | *0.88* |
| ALT/ GPT (on discharge) | 63.02 ± 102.69 \| 39 (5-1168) | 61.87 ± 85.06 \| 39.96 (5-942) | 73.20 ± 201.91 \| 29.50 (9-1168) | *0.55* |
| AST/ GOT (on admission) | 115.48 ± 377.37 \| 31 (5-4238) | 104.44 ± 353.48 \| 31 (5-4238) | 216.77 ± 547.34 \| 35 (9-2152) | *0.09* |
| AST/ GOT (day 3) | 127.00 ± 532.03 \| 30.50 (4-5549) | 105.47 ± 423.85 \| 29.50 (4-5246) | 334.05 ± 1129.18 \| 41 (12-5549) | ***0.04*** |
| AST/ GOT (day 7) | 63.00 ± 122.46 \| 34 (6-1150) | 60.84 ± 120.02 \| 34 (6-1150) | 81.70 ± 143.46 \| 32.25 (11-595.20) | *0.43* |
| AST/ GOT (on discharge) | 72.80 ± 381.57 \| 29 (5-6300) | 51.64 ± 93.39 \| 30 (5-988) | 258.83 ± 1162.99 \| 24 (5-6300) | ***0.005*** |
| Transaminitis (AST or ALT >40 UI·L^-1^) | 58.01 % (257) | 58.00 % (232) | 58.14 % (25) | *0.24* |
| Bilirrubin (on admission) | 0.93 ± 1.45 \| 0.59 (0.09-19.27) | 0.92 ± 1.50 \| 0.58 (0.10-19.72) | 0.95 ± 0.87 \| 0.70 (0.09-4.04) | *0.93* |
| Bilirrubin (day 3) | 0.95 ± 1.86 \| 0.51 (0.07-25) | 0.94 ± 1.91 \| 0.50 (0.07-25) | 1.11 ± 1.13 \| 0.71 (0.10-6.20) | *0.62* |
| Bilirrubin (day 7) | 0.81 ± 1.24 \| 0.50 (0.08-12.65) | 0.78 ± 1.29 \| 0.47 (0.08-12.65) | 0.97 ± 0.71 \| 0.77 (0.09-2.84) | *0.42* |
| Bilirrubin (on discharge) | 0.80 ± 1.18 \| 0.50 (0.06-9.85) | 0.74 ± 1.10 \| 0.49 (0.06-9.85) | 1.34 ± 1.70 \| 0.71 (0.09-8.00) | ***0.008*** |
| High bilirrubin levels (>21μmol·L^-1^/ >1.23 mg·dL^-1^) | 25.06 % (111) | 23.50 % (94) | 39.53 % (17) | *0.89* |
| ALP (on admission) | 88.40 ± 62.66 \| 72 (21-620) | 88.07 ± 63.90 \| 71 (21-620) | 91.53 ± 50.34 \| 75 (42-274) | *0.78* |
| ALP (day 3) | 96.86 ± 67.09 \| 77.50 (22-644) | 93.91 ± 63.34 \| 76.50 (22-644) | 126.62 ± 93.83 \| 104 (33-476) | ***0.02*** |
| ALP (day 7) | 130.16 ± 127.83 \| 96 (34-1080) | 129.53 ± 132.65 \| 94 (34-1080) | 135.85 ± 73.71 \| 106.50 (42-304) | *0.83* |
| ALP (on discharge) | 127.57 ± 106.74 \| 97.50 (6-740) | 123.41 ± 97.33 \| 97 (32-724) | 163.23 ± 165.36 \| 102 (6-740) | *0.06* |
| High ALP levels (>129 UI·L^-1^) | 31.38 % (139) | 30.50 % (122) | 39.53 % (17) | *0.36* |
| GGT (on admission) | 90.30 ± 141.31 \| 42 (5-1037) | 87.87 ± 139.60 \| 40 (7-1037) | 112.29 ± 156.75 \| 66 (5-817) | *0.37* |
| GGT (day 3) | 110.11 ± 177.97 \| 60.50 (4-1838) | 104.78 ± 172.62 \| 57 (4-1838) | 162.77 ± 221.43 \| 82 (12-945) | *0.12* |
| GGT (day 7) | 183.47 ± 194.84 \| 116 (10-1301) | 185.03 ± 201.37 \| 116 (10-1301) | 171.17 ± 134.83 \| 119 (47-553) | *0.73* |
| GGT (on discharge) | 172.01 ± 211.74 \| 103 (8-2051) | 172.65 ± 213.39 \| 104 (11-2051) | 166.84 ± 201.05 \| 88 (8-751) | *0.88* |
| High GGT levels (>67 UI·L^-1^) | 63.88 % (283) | 62.50 % (250) | 76.74 % (33) | *0.47* |

ALT: Alanine transaminase; GPT: glutamate-pyruvate transaminase; AST: aspartate aminotransferase; GOT: glutamic oxaloacetic transaminase; ALP: Alkaline phosphatase; GGT: Gamma-glutamyltransferase.

| **D** | All patients  (n=443) | EN  (n=400) | EN with PN  (n=43) | *P* |
| --- | --- | --- | --- | --- |
| ***Haematology*** | | | | |
| Leukocytes (on admission) | 13.41 ± 6.67 \| 12.53 (0-40.08) | 13.34 ± 6.55 \| 12.35 (0.40-40.08) | 14.16 ± 7.83 \| 14.20 (0-30.40) | *0.46* |
| Leukocytes (day 3) | 12.06 ± 5.87 \| 11.16 (0.10-39.80) | 12.03 ± 5.82 \| 11.13 (0.30-39.80) | 12.27 ± 6.40 \| 11.20 (0.10-22.70) | *0.81* |
| Leukocytes (day 7) | 12.11 ± 5.42 \| 11.50 (0.10-30.40) | 12.08 ± 5.25 \| 11.44 (1.40-30.40) | 12.33 ± 6.70 \| 11.80 (0.10-25.63) | *0.79* |
| Leukocytes (on discharge) | 10.98 ± 5.67 \| 10 (0.10-46.44) | 10.88 ± 5.37 \| 10.00 (0.10-46.44) | 11.99 ± 8.03 \| 10.29 (0.10-31.63) | *0.24* |
| Leukocytosis (>11000) | 77.88 % (345) | 77.50 % (310) | 81.40 % (35) | ***0.006*** |
| Leukopenia (<3900) | 8.80 % (39) | 7.75 % (31) | 18.60 % (8) | *0.09* |
| Lymphocyte (on admission) | 1.38 ± 1.21 \| 1.00 (0-6.80) | 1.41 ± 1.23 \| 1.01 (0.10-6.80) | 1.14 ± 0.87 \| 0.98 (0-4.10) | *0.18* |
| Lymphocyte (day 3) | 1.29 ± 1.10 \| 1.00 (0-8.60) | 1.32 ± 1.13 \| 1.00 (0-8.60) | 0.98 ± 0.57 \| 0.90 (0-2.80) | *0.06* |
| Lymphocyte (day 7) | 1.40 ± 1.08 \| 1.17 (0.05-7.20) | 1.43 ± 1.09 \| 1.20 (0.10-7.20) | 1.10 ± 0.94 \| 0.94 (0.05-5.00) | *0.09* |
| Lymphocyte (on discharge) | 1.47 ± 1.07 \| 1.23 (0-8.30) | 1.51 ± 1.06 \| 1.27 (0.10-8.30) | 1.18 ± 1.13 \| 0.94 (0-6.90) | *0.07* |
| Lymphopenia (<1300) | 88.49 % (392) | 88.25 % (353) | 90.70 % (39) | *0.21* |
| Platelets (on admission) | 208.08 ± 108.61 \| 195 (5-1000) | 209.75 ± 105.95 \| 197 (7-1000) | 192.51 ± 131.20 \| 168 (5-624) | *0.33* |
| Platelets (day 3) | 186.45 ± 104.05 \| 172 (2-924) | 187.71 ± 103.31 \| 175.50 (2-924) | 175.05 ± 111.19 \| 159 (10-415) | *0.45* |
| Platelets (day 7) | 228.87 ± 120.95 \| 214 (9-742) | 235.80 ± 121.75 \| 219 (9-742) | 173.58 ± 99.57 \| 172 (18-497) | ***0.003*** |
| Platelets (on discharge) | 291.52 ± 171.63 \| 267 (0.70-979) | 295.88 ± 170.06 \| 267 (0.70-979) | 249.83 ± 183.06 \| 267.50 (5-899) | *0.10* |
| Low plasma platelets (<100000) | 23.02 % (102) | 21.00 % (84) | 41.86 % (18) | *0.98* |
| ***Renal*** | | | | |
| Creatinine (on admission) | 1.42 ± 1.28 \| 1.00 (0.22-9.64) | 1.37 ± 1.29 \| 0.97 (0.22-9.64) | 1.83 ± 1.14 \| 1.50 (0.38-4.69) | ***0.03*** |
| Creatinine (day 3) | 1.21 ± 1.21 \| 0.81 (0.25-11.14) | 1.19 ± 1.22 \| 0.79 (0.25-11.14) | 1.46 ± 1.11 \| 1.05 (0.35-4.17) | *0.17* |
| Creatinine (day 7) | 1.13 ± 1.19 \| 0.73 (0.18-12.75) | 1.08 ± 1.18 \| 0.70 (0.18-12.75) | 1.49 ± 1.18 \| 1.19 (0.33-6.00) | *0.05* |
| Creatinine (on discharge) | 0.96 ± 1.01 \| 0.65 (0.15-9.51) | 0.92 ± 0.99 \| 0.63 (0.15-9.51) | 1.26 ± 1.14 \| 0.77 (0.28-6.30) | ***0.04*** |
| Renal failure (Creatinine >100 μmol·L^-1^) | 56.88 % (252) | 55.00 % (220) | 74.42 % (32) | ***0.04*** |
| ***Blood Proteins*** | | | | |
| Prealbumin (on admission) | 158.01 ± 77.96 \| 156 (21-473) | 158.62 ± 74.56 \| 157 (30-473) | 152.50 ± 106.55 \| 126 (21-450) | *0.76* |
| Prealbumin (day 3) | 151.20 ± 77.25 \| 138.50 (22-450) | 150.95 ± 75.62 \| 139 (22-450) | 153.84 ± 95.58 \| 130 (46-340) | *0.89* |
| Prealbumin (day 7) | 201.24 ± 106.83 \| 182.50 (18.70-517) | 209.82 ± 106.66 \| 195 (18.70-517) | 133.67 ± 83.49 \| 122.50 (20-350) | ***0.007*** |
| Prealbumin (on discharge) | 206.38 ± 92.14 \| 194.50 (57-430) | 211.76 ± 93.01 \| 205 (57-430) | 160.13 ± 71.06 \| 160 (60-327) | ***0.04*** |
| Low prealbumin levels (<200 mg·L^-1^) | 51.92 % (230) | 51.50 % (206) | 55.81 % (24) | ***0.03*** |
| Albumin (on admission) | 3.07 ± 0.65 \| 3.10 (1-5) | 3.10 ± 0.62 \| 3.10 (1.55-5.00) | 2.71 ± 0.79 \| 2.50 (1-4.80) | ***0.001*** |
| Albumin (day 3) | 2.80 ± 0.56 \| 2.80 (1.18-4.40) | 2.84 ± 0.56 \| 2.90 (1.18-4.40) | 2.44 ± 0.48 \| 2.30 (1.83-3.50) | ***<0.001*** |
| Albumin (day 7) | 2.75 ± 0.58 \| 2.80 (1.16-4.70) | 2.79 ± 0.57 \| 2.80 (1.16-4.70) | 2.33 ± 0.42 \| 2.40 (1.64-3.30) | ***<0.001*** |
| Albumin (on discharge) | 2.93 ± 0.62 \| 3.00 (1.20-4.40) | 2.98 ± 0.60 \| 3.00 (1.20-4.40) | 2.55 ± 0.64 \| 2.45 (1.70-3.90) | ***<0.001*** |
| Low albumin levels (<30 g·L^-1^) | 95.03 % (421) | 94.75 % (379) | 97.67 % (42) | ***<0.001*** |
| ***C-Reactive Protein levels*** | | | | |
| C-Reactive Protein (on admission) | 114.79 ± 125.70 \| 64.20 (0.01-609.60) | 110.02 ± 123.62 \| 59.00 (0.01-609.60) | 160.24 ± 137.91 \| 149.00 (0.07-472) | *0.07* |
| C-Reactive Protein (day 3) | 123.82 ± 130.82 \| 75.00 (0.01-588.80) | 119.99 ± 127.37 \| 74.50 (0.01-588.80) | 157.43 ± 156.25 \| 112.40 (0.38-533) | *0.12* |
| C-Reactive Protein (day 7) | 100.56 ± 111.26 \| 61.00 (0.40-600) | 97.28 ± 112.29 \| 55.90 (0.40-600) | 127.75 ± 100.26 \| 126.50 (0.80-371) | *0.18* |
| C-Reactive Protein (on discharge) | 69.59 ± 83.89 \| 37.60 (0.20-478) | 64.11 ± 77.26 \| 35.60 (0.20-478) | 116.84 ± 119.17 \| 63.50 (0.90-400.50) | ***0.001*** |

**Figure S1.** Algorithm to select the feeding route for giving nutrition therapy in ICU patients [13, 15].
